# Transcriptomics Reveals the Effect of Short-Term Freezing on the Signal Transduction and Metabolism of Grapevine

**DOI:** 10.3390/ijms24043884

**Published:** 2023-02-15

**Authors:** Xing Han, Yi-Han Li, Mo-Han Yao, Fei Yao, Zhi-Lei Wang, Hua Wang, Hua Li

**Affiliations:** 1 College of Enology, Northwest A&F University, Xianyang 712100, China; 2 College of Plant Protection, Northwest A&F University, Xianyang 712100, China; 3 China Wine Industry Technology Institute, Yinchuan 750021, China; 4 Shaanxi Engineering Research Center for Viti-Viniculture, Xianyang 712100, China

**Keywords:** *Vitis vinifera* L., freezing tolerance, RNA sequencing, differentially expressed genes (DEGs), signal transduction, carbohydrate metabolism

## Abstract

Low temperature is an important factor limiting plant growth. Most cultivars of *Vitis vinifera* L. are sensitive to low temperatures and are at risk of freezing injury or even plant death during winter. In this study, we analyzed the transcriptome of branches of dormant cv. Cabernet Sauvignon exposed to several low-temperature conditions to identify differentially expressed genes and determine their function based on Gene Ontology (GO) and Kyoto Encyclopedia of Genes and Genomes (KEGG)enrichment analyses. Our results indicated that exposure to subzero low temperatures resulted in damage to plant cell membranes and extravasation of intracellular electrolytes, and that this damage increased with decreasing temperature or increasing duration. The number of differential genes increased as the duration of stress increased, but most of the common differentially expressed genes reached their highest expression at 6 h of stress, indicating that 6 h may be a turning point for vines to tolerate extreme low temperatures. Several pathways play key roles in the response of Cabernet Sauvignon to low-temperature injury, namely: (1) the role of calcium/calmodulin-mediated signaling; (2) carbohydrate metabolism, including the hydrolysis of cell wall pectin and cellulose, decomposition of sucrose, synthesis of raffinose, and inhibition of glycolytic processes; (3) the synthesis of unsaturated fatty acids and metabolism of linolenic acid; and (4) the synthesis of secondary metabolites, especially flavonoids. In addition, pathogenesis-related protein may also play a role in plant cold resistance, but the mechanism is not yet clear. This study reveals possible pathways for the freezing response and leads to new insights into the molecular basis of the tolerance to low temperature in grapevine.

## 1. Introduction

Low temperature is one of the main factors limiting the growth and distribution of plants. Plants have evolved sophisticated mechanisms to withstand cold stress, such as cold acclimation [1,2]. The sensing of low temperature and the transmission of signals by plants are essential for their resistance to cold [3]. Low temperature first changes the structure of the cell membrane from a liquid phase to a solid phase, and the hardening of the cell membrane leads to changes in the actin backbone, which triggers the activation of the calcium signaling pathway [4] and alters the function of membrane lipids and membrane proteins to transmit low-temperature signals [5]. The intracellular Ca^2+^ level is the second messenger for plants to sense cold signals. The rapid increase in the intracellular Ca^2+^ concentration is decoded and delivered to the plant by calcium-binding proteins to activate the expression of transcription factors [6]. There are a variety of Ca^2+^ receptors in plants; Calmodulin (CaM) and Calmodulin-like (CML) are the two most studied classes, and their binding to Ca^2+^ alters the enzymatic activity or structure of their interacting proteins to activate transcriptional, protein phosphorylation, or metabolic changes to regulate plant resistance [7,8,9].

Changes in phosphorylation induced by the mitogen-activated protein kinase (MAPK) cascade reaction are considered to be another common plant low-temperature signal pathway [10]. In Arabidopsis, low-temperature stress induces the expression of *MKK2*, which activates *MAPK4/MAPK5*, thereby inhibiting the activity of *MAPK3/MAPK6*, then reducing the phosphorylation level of *ICE1*, increasing the stability and transcriptional activity of *ICE1,* and improving plant cold resistance [11,12,13]. In addition to the positive regulation of *CBFs* by *ICE1*, the expression of the *CBF* gene is also negatively regulated by *MYB15*, which is a transcription inhibitor of cold signals, and its activity is also regulated by *MPK6*-mediated phosphorylation [14].

Other messenger molecules such as reactive oxygen species (ROS) are also involved in regulating plant responses to cold stress. ROS play a dual role in plant cells, they can induce gene expression and protein synthesis to protect cells from stress, and on the other hand, they can induce oxidative stress [15]. Flavonoids are currently widely recognized for their antioxidant function in plant abiotic stress, being effective scavengers of ROS, inhibiting reactive oxygen species’ production and maintaining H_2_O_2_ concentrations in the sublethal concentration range [16]. Changes in cellular redox homeostasis activate flavonoid biosynthesis, particularly flavonol metabolism [17], and severe stress conditions may inactivate antioxidant enzymes while upregulating flavonol biosynthesis [18,19]. 

At low temperatures below the freezing point, cell water is transformed into extracellular ice, leading to cell dehydration and cell structure disintegration [20,21], which is also a way of perceiving low temperatures. Plants reduce cell volume shrinkage and prevent cell deformation by increasing the thickness and strength of cell walls [22]. At low temperature, homogalacturonic acid of pectin undergoes demethylation, releases methanol and protons, and generates negatively charged carboxyl groups in this process [23]. These molecules with galacturonic acid residues will bind to Ca^2+^ to form dimers, also known as the “egg box” structure, which is the basis for the formation of pectin gels [24]. Pectin can control cell wall porosity and cell adhesion [25,26], thus preventing ice diffusion and promoting the supercooling of intracellular water under low temperature or freezing stress [27]. 

Grapes are one of the most widely cultivated fruits in the world and have an important economic value. *Vitis vinifera* L. varieties are widely cultivated for their excellent fruit quality, but they are very sensitive to cold and are at risk of cold damage or even death in winter, especially in some cold wine regions such as northern China [28], northeastern Canada [29], northeastern United States [30], northern Europe [31], and the Russian Far East [32]. Cross breeding can provide germplasm resources with high resistance to cold and are considered the most fundamental solution. However, studies have shown that cold resistance in grapes is a complex trait controlled by micro-effective polygenes, and the major cold-resistance genes in wild species appear to have a chain reaction, with their genes controlling undesirable fruit quality traits [33,34]. Therefore, it is an effective strategy to screen and use the cold resistance-related genes of *V. vinifera* L. for intraspecific hybridization and molecular breeding to obtain high-quality and cold resistant cultivars. Exploring how *V. vinifera* L. responds to low temperatures, especially extreme temperatures below freezing, can help us understand the mechanism of cold resistance and screen key genes to evaluate cold resistance and improve germplasm.

Therefore, the objective of this study was to identify transcripts and pathways that are differentially expressed in dormant branch tissue and enriched in response to various freezing stresses. Six groups of samples at different temperatures and times were detected to reveal the transcriptional landscape, and the key genes and pathways were analyzed to propose a hypothetical model of how grapevine responds to low-temperature stress.

## 2. Results

### 2.1. Selection of Experimental Conditions

**Figure 1:**
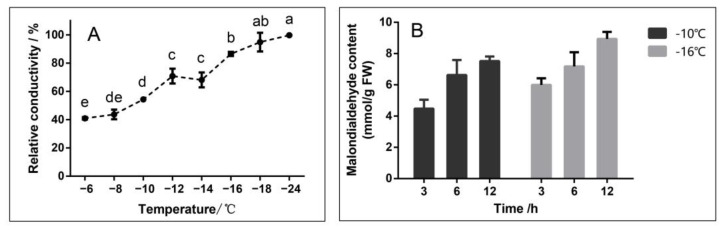
Relative conductivity (**A**) and malondialdehyde content (**B**). (**A**): Relative conductivity of branches after 12 h treatment at different temperatures; means with the same lowercase letter are not significantly different among the different temperatures according to Tukey’s test (*p* ≤ 0.05); (**B**): Malondialdehyde (MDA) content of branches under different stress times at −10 °C and −16 °C.

The electrolyte leakage of dormant cuttings branches was first measured in this study (Figure 1A), and two temperatures with relative conductivities close to 50% (−10 °C) and 80% (−16 °C) were selected for the next analysis. Based on our previous study [35], three time points of 3, 6, and 12 h of low-temperature stress were identified as sampling points. The malondialdehyde (MDA) content of branches at three time points was measured. It can be seen that the content of MDA increased with the extension of the stress time, and the content of MDA at −16 °C at the same time point was higher than that at −10 °C (Figure 1B), indicating that the oxidative damage of cells was more serious with the deepening of stress.

### 2.2. Expression Analysis of the Data Set

**Figure 2:**
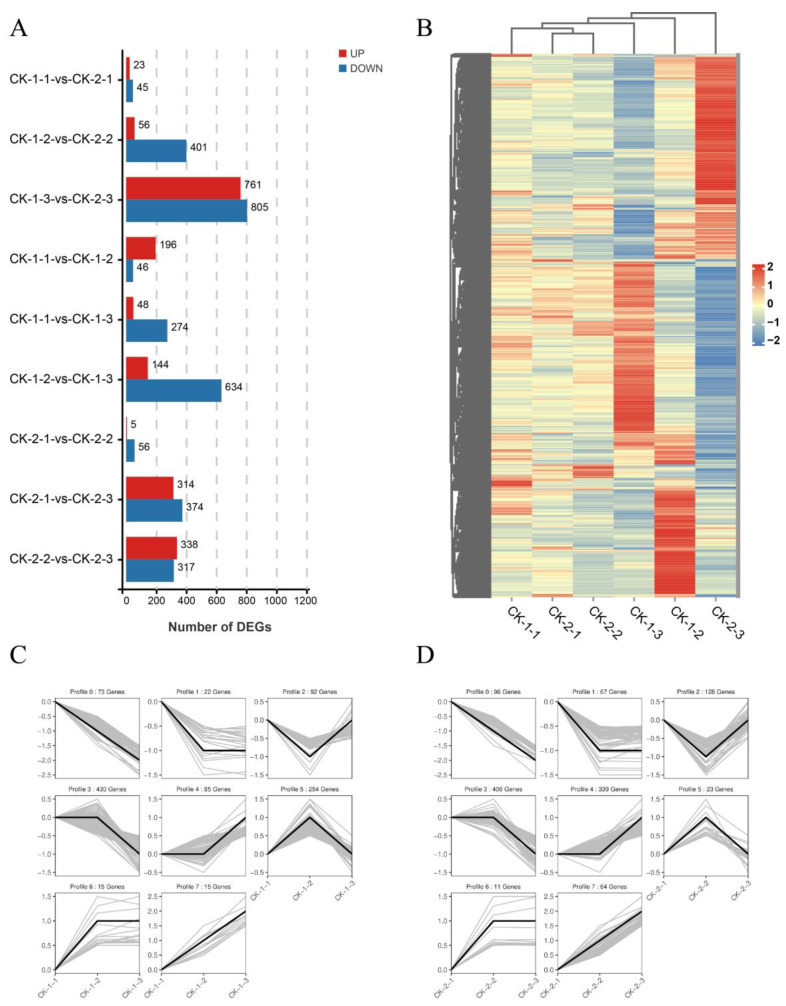
Overall analysis of differential gene expression between different treatment groups. (**A**): number of differentially expressed genes (DEGs) between different groups. (**A**) vs. (**B**) indicates the expression of (**B**) relative to (**A**). Red indicates up-regulation, blue indicates down-regulation. The screening conditions for differential genes were |log2FC| ≥ 1 and FDR < 0.05; B: Heat map of gene expression in different treatment groups. Each column represents one sample, and each row represents one gene. Gene expressions of rows were normalized using the z-score; the more red the color, the higher the gene expression, the more blue the color, the lower the gene expression; (**C**): The trend pattern of DEGs in the groups under −10 °C stress (CK-1-1 vs. CK-1-2 vs. CK-1-3) with time. The black line represents the trend line, and the gray line represents each gene; (**D**): The trend pattern of DEGs in the groups under −16 °C stress (CK-2-1 vs. CK-2-2 vs. CK-2-3) over time.

All transcripts that were significantly differentially expressed at least in one time-point between treatment groups were extracted, yielding a total of 2220 differentially expressed genes (DEGs), and divided into nine different groups (Figure 2A). The largest number of these differential genes was expressed between CK-1-3 and CK-2-3, which indicated that the different stress temperatures greatly affected the transcription of the vines. In addition, regardless of temperature, the number of differential genes was higher at the 6 h and 12 h time points than at 3 h, indicating that more genes were differentially expressed as the stress time increased. Further analysis of the DEGs between groups showed that the gene expression patterns of CK-1-3, CK-1-2, and CK-2-3 differed significantly, suggesting that 6 h may be an important turning point, with a significant change from the short cold treatment (3 h) (Figure 2B).

The DEG data at different times were normalized according to the trend, and the pattern of each trend was drawn. In total, 979 differential genes were obtained in the CK-1-1 vs. CK-1-2 vs. CK-1-3 group, which were divided into seven trend groups (Profile) (Figure 2C), where Profile 3 had the highest number of differential genes (430), followed by Profile 5 (254), and these groups also differed significantly (*p* < 0.05). The CK-2-1 vs. CK-2-2 vs. CK-2-3 group showed 1185 differential genes divided into seven trend groups (Profiles) (Figure 2D), among which Profile 3 also had the highest number of differential genes (451) and the most significant differences. This indicated that the expression trends of genes over time were basically the same at different temperatures, and the genes in Profile 3 were further analyzed.

### 2.3. Functional Analyses of the Transcriptome Changes in Response to Different Freezing Treatments

**Figure 3:**
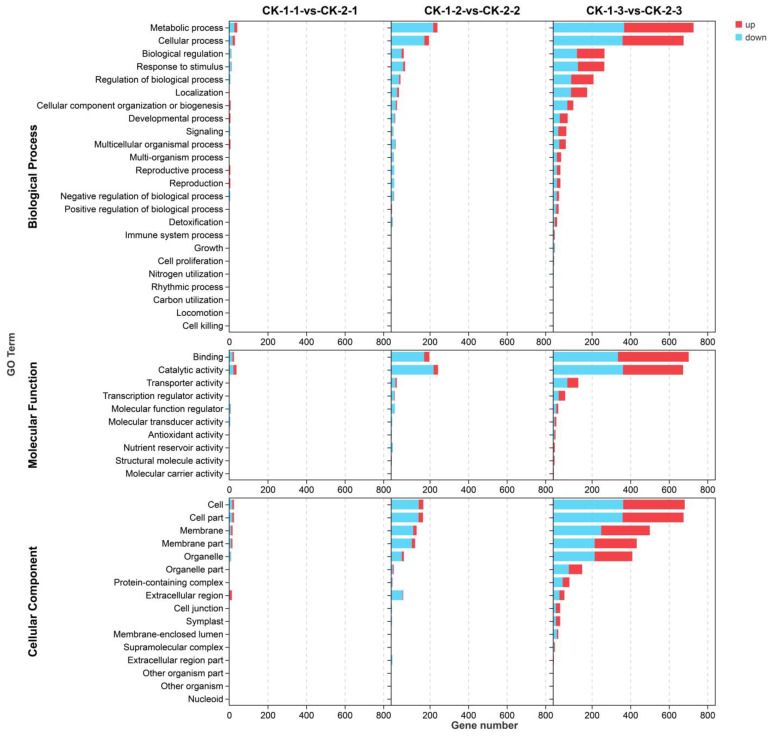
Gene Ontology (GO) functional annotation analysis of DEGs in groups under different stress temperatures.

Gene Ontology (GO) enrichment analysis was performed for DEGs at three time points under different freezing treatments and assigned to three major categories (Figure 3). The different stress temperatures had the highest number of differential genes at 12 h, and therefore this time was also enriched in the most abundant GO term. In the biological process category, the DEGs in the two comparison groups CK-1-1 vs. CK-2-1 and CK-1-2 vs. CK-2-2 were mainly enriched in the two GO terms of metabolic process (GO:0008152) and cellular process (GO:0008152), and the DEGs in the CK-1-3 vs. CK-2-3 group were also enriched in GO terms that included response to stimulus (GO:0008152), biological regulation (GO:0065007), and regulation of biological process (GO:0065007). In the molecular function category, the differential genes in the CK-1-1 vs. CK-2-1 and CK-1-2 vs. CK-2-2 comparison groups were mainly enriched in the GO terms of binding (GO:0005488) and catalytic activity (GO:0003824), while the differential genes in the CK-1-3 vs. CK-2-3 group were also enriched in the GO terms of transporter activity (GO:0005215) and transcription regulator activity (GO:0140110). Further, cell (GO:0005623), cell part (GO:0043226), membrane (GO:0016020), membrane part (GO:0044425), and organelle (GO:0043226) were the top five GO terms of the cellular component category in the three groups. The extracellular region (GO:0005576) was also the GO term with enrichment of many DEGs in the two comparison groups: CK-1-1 vs. CK-2-1 and CK-1-2 vs. CK-2-2. The GO enrichment of differential genes in Profile 3 was essentially the same at both temperatures, and the top two enriched GO terms in the three categories were also metabolic process, cellular process, binding, catalytic activity, cell, and cell part (Figure S1).

Kyoto Encyclopedia of Genes and Genomes (KEGG) enrichment analysis was performed for the DEGs in each group; all nine comparison groups showed significant differences in metabolic pathways (ko01100) and alpha-linolenic acid metabolism (ko00592), and other pathways with significant differences were also annotated (Table S1). The pathways that showed significant differences in more than half of the comparison groups were biosynthesis of secondary metabolites (ko01110) (8, being significant in eight comparison groups, the same below), pentose and glucuronate interconversions (ko00040) (8), flavonoid biosynthesis (ko00941) (6), circadian (ko04712) (5), and linoleic acid metabolism (ko00591) (5). In addition, pathways related to amino acid metabolism (ko00270, ko00350, ko00460, ko00520, ko00360), secondary metabolite synthesis (ko00945, ko00960, ko00940, ko00073), sugar metabolism (ko00010, ko00052), and signal transduction (ko04075, ko04016) were also enriched several times.

**Figure 4:**
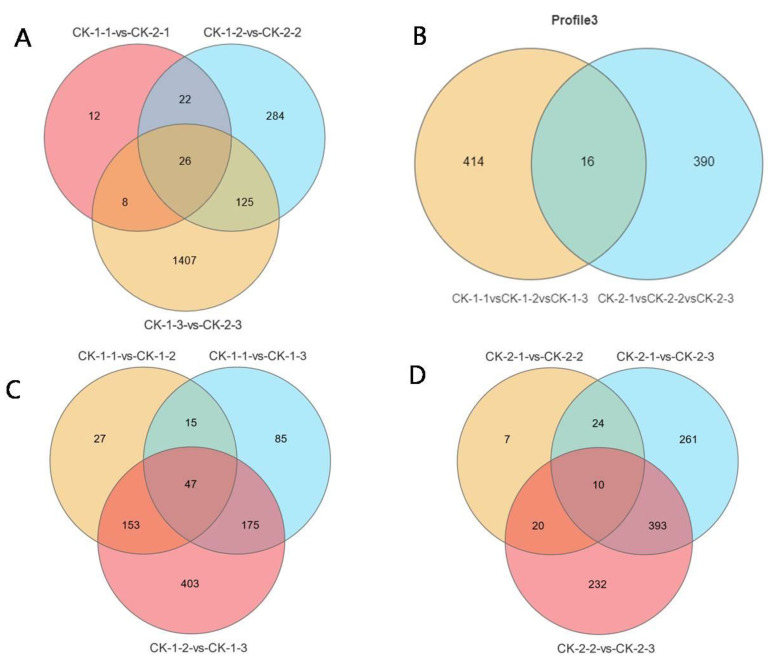
Expression of common differential genes (DEGs) in groups under different freezing treatments. (**A**): Venn diagrams of the changes in DEGs between the two temperatures at the same time; (**B**): Venn diagrams of the changes in the DEGs of Profile 3 under the two temperatures; (**C**): Venn diagrams of the changes in DEGs between different times at −10 °C; (**D**): Venn diagrams of the changes in DEGs between different times at −16 °C.

A Venn diagram analysis of the three comparison groups (Figure 4A) revealed that 26 DEGs (1.38%) were co-expressed under different stress temperatures. The number of differential genes specific to each of the three comparison groups varied greatly, with only 12 DEGs specifically expressed at 3 h, 284 DEGs specifically expressed at 6 h, and 1407 DEGs specifically expressed at 12 h. KEGG enrichment analysis of common DEGs under different temperatures showed (Table 1A) that they were enriched in 14 pathways affecting signal transduction (plant–pathogen interaction, circadian rhythm—plant), carbohydrates (pentose and glucuronate interconversions), lipids (linoleic acid metabolism, alpha-linolenic acid metabolism, fatty acid elongation, amino sugar and nucleotide sugar metabolism), amino acids metabolism (biosynthesis of amino acids, cysteine and methionine metabolism), and secondary metabolite biosynthesis (flavonoid biosynthesis, stilbenoid, diarylheptanoid and gingerol biosynthesis, sesquiterpenoid and triterpenoid biosynthesis).

Venn diagram analysis was also performed to take the intersection of differential genes for each comparison group at different times (Figure 4B–D). There were 47 common DEGs for the three comparison groups at −10 °C (CK-1-1 vs. CK-1-2, CK-1-1 vs. CK-1-3 and CK-1-2 vs. CK-1-3), 10 common DEGs for the three comparison groups at −16 °C (CK-2-1 vs. CK-2-2, CK-2-1 vs. CK-2-3 and CK-2-2 vs. CK-2-3), and 16 common DEGs for two Profile 3 groups. The three groups of co-differential genes were taken together and further analyzed to obtain 21 functionally annotated genes, 15 of which were annotated to pathways by KEGG enrichment analysis, affecting signal transduction (plant–pathogen interaction, plant hormone signal transduction, MAPK signaling pathway—plant, phosphatidylinositol signaling system, circadian rhythm—plant), the metabolism of lipids (fatty acid metabolism, fatty acid biosynthesis, biosynthesis of unsaturated fatty acids, alpha-linolenic acid metabolism), carbohydrates (carbon metabolism, glycolysis/gluconeogenesis, carbon fixation in photosynthetic organisms, starch and sucrose metabolism, pentose and glucuronate interconversions, galactose metabolism), amino acids (biosynthesis of amino acids, cyanoamino acid metabolism, cysteine and methionine metabolism), and the synthesis of secondary metabolites (phenylpropanoid biosynthesis, 2-oxocarboxylic acid metabolism, flavonoid biosynthesis, sesquiterpenoid and triterpenoid biosynthesis) (Table 1B).

### 2.4. Response of Cabernet Sauvignon to Low Temperatures

Based on the KEGG enrichment analysis, four categories, i.e., signal transduction, carbohydrate metabolism, lipid metabolism, and secondary metabolite synthesis, were further analyzed to explain the gene expression pattern of Cabernet Sauvignon in response to low-temperature signals.

#### 2.4.1. Effect of Freezing Stress on Signal Transduction

**Figure 5:**
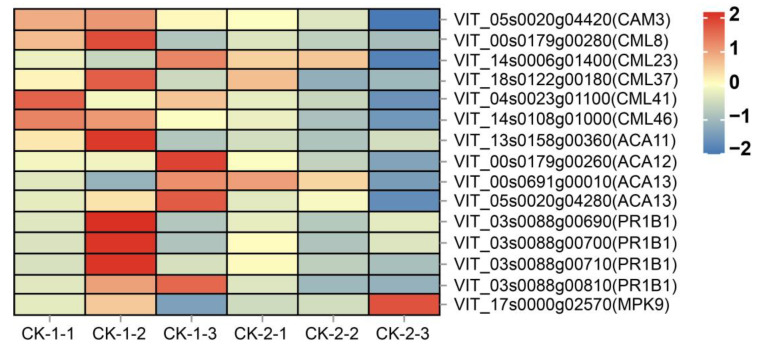
Heatmap of gene expression related to signal transduction.

The genes that were differentially expressed under different stress conditions included one *CAM* gene, five *CML* genes, and four *ACA* (calcium-transporting ATPase) (Figure 5). Overall, the expression of Ca^2+^ signaling-related genes was higher under −10 °C stress, probably due to the extreme low-temperature stress at −16 °C that disturbed the physiological and biochemical metabolism. In addition, cells started to undergo apoptosis, and calcium receptors were inactive. Further analysis of the three time points under −10 °C stress showed that the expression of the *CML41* and *CML46* genes reached the highest value at 3 h and then gradually decreased; the *CML8*, *CML37*, and *CAM3* genes showed high expression at 6 h that decreased at 12 h, while the *ACA12*, and *ACA13* gene expression reached the highest value at 12 h. This indicated that the instantaneous increase in Ca^2+^ was sensed first by calcium receptors at the early stage of cold stress, and calcium/calmodulin-mediated signaling may play a key role in the response to cold stress.

*CML8*, as a common differential gene, affected four pathways, i.e., plant–pathogen interaction, phytohormone signaling, MAPK signaling pathway—plant, and phosphatidylinositol signaling system (Table 1). The up-regulation of PR1B1-encoding genes was also observed, with the highest expression at 6 h (*VIT_03s0088g00690*, *VIT_03s0088g00700*, *VIT_03s0088g00710*) and 12 h (*VIT_03s0088g00810*) (Figure 5). Seven MAPK-related genes were identified in this study, but only *MPK9* showed differences between treatments (Figure 5). 

#### 2.4.2. Effect of Freezing Stress on Sugar Metabolism

**Figure 6:**
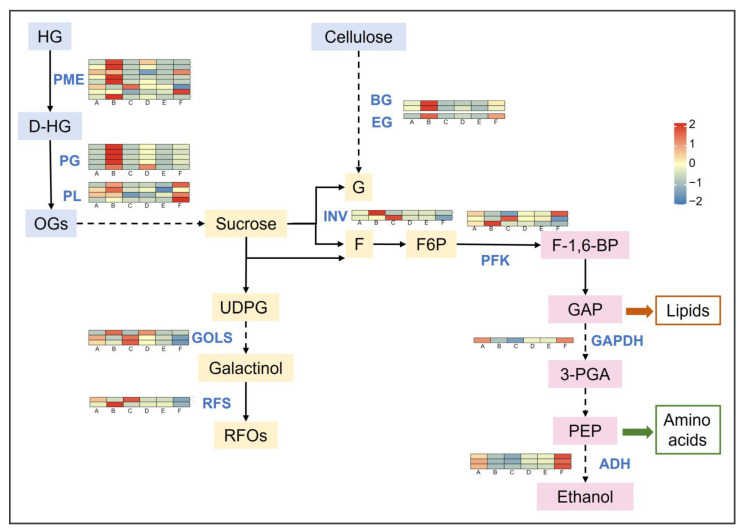
Carbohydrate metabolic pathways in Cabernet Sauvignon plants under freezing stress. Note: The dotted line indicates that there is no direct conversion relationship between the two compounds, and several steps are omitted. A: CK-1-1; B: CK-1-2; C: CK-1-3; D: CK-2-1; E: CK-2-2; F: CK-2-3. HG: homogalacturonic acid; D-HG: demethylated homogalacturonic acid; OGs: oligogalacturonic acid; G: glucose; F: fructose; F6P: fructose-6-phosphate; F-1,6-BP: fructose-1,6-bisphosphate; GAP: glyceraldehyde- 3-phosphate; 3-PGA: 3-phosphoglyceric acid; PEP: phosphoenolpyruvate; UDPG: uridine diphosphate glucose; RFOs: raffinose. PME: pectinesterase, EC:3.1.1.11; PG: polygalacturonase, E3.2.1.15; PL: pectate lyase, EC:4.2.2.2; BG: beta-glucosidase, E3.2.1.21; EG: endoglucanase, E3.2.1.4; SUS: sucrose synthase, E2.4.1.13; GOLS: inositol 3-alpha-galactosyltransferase, EC:2.4.1.123; RFS: raffinose synthase, EC2.4.1.82; INV: Invertase, EC:3.2.1.26; PFK: 6-phosphofructokinase, EC:2.7.1.11; GAPDH: glyceraldehyde 3-phosphate dehydrogenase, EC:1.2.1.12; ADH: alcohol dehydrogenase, EC:1.1.1.1.

KEGG enrichment of common DEGs showed significant differences in pentose and glucuronide interconversion pathways at different stress temperatures, mainly affecting polygalacturonase (PG) activity (Table 1A), while differential genes were enriched in six related pathways at different stress times, i.e., carbon metabolism, glycolysis/gluconeogenesis, carbon sequestration by photosynthetic organisms, starch and sucrose metabolism, pentose and glucuronide interconversion, and galactose metabolism (Table 1B), indicating that carbohydrates play an important role in grapevines in response to sustained low-temperature stress. The sugar metabolic pathways of plants under freezing stress were analyzed, including the hydrolysis of cell wall polysaccharides, the conversion of sucrose and starch, the synthesis of raffinose, and the glycolysis process (Figure 6).

Pectinesterase (PME) catalyzes the specific demethylation of homogalacturonan (HG) in cell wall pectin, which facilitates pectin gel formation and cell wall rigidity. Under low-temperature stress, most of the eight genes encoding PME (*VIT_06s0009g02560*, *VIT_06s0009g02590*, *VIT_15s0048g00510*, *VIT_16s0022g00700*, *VIT_07s0005g00720*) showed the highest expression at CK-1-2 (B) and then decreased substantially (C vs. B), and lower stress temperatures (E vs. B) also caused a decrease in PME gene expression. The partially demethylated HG was further depolymerized by pectin-degrading enzymes, such as polygalacturonase (PG) and pectate lyase (PL), producing more oligogalacturonic acid (OG) and monomeric galacturonides to provide a carbon source for the cells. Cellulose in the cell wall is also hydrolyzed into soluble sugars by β-glucosidase (BG) and endoglucanase (EG), and the genes encoding them (*VIT_06s0004g01430*, *VIT_06s0004g01440*, *VIT_18s0089g00210*) also had the highest expression in CK-1-2 (B).

Low temperature also stimulated the degradation of sucrose, including the hydrolysis of β-D-ribofuranosidic bonds catalyzed by invertase (INV) to produce glucose (G) and fructose (F), and the production of fructose (F) and uridine diphosphate glucose (UDPG) catalyzed by sucrose synthase (SUS). UDPG was converted to galactinol by the action of inositol 3-alpha-galactosyltransferase (GOLS) and then catalyzed by raffinose synthase (RFS) to produce raffinose series oligosaccharides (RFOs), which have multiple functions in plant responses to abiotic stresses. The two genes encoding INV (*VIT_16s0022g00670*, *VIT_09s0002g02320*) showed the highest expression at CK-1-2 and CK-1-3, respectively, and the expression of SUS-encoded genes (*VIT_17s0053g00700*, *VIT_04s0079g00230*) also reached the highest at CK-1-2; both of them can promote sucrose catabolism in plants at low temperatures. The expressions of the GOLS (*VIT_14s0060g00760*, *VIT_14s0060g00790*) and RFS (*VIT_17s0000g08960*) genes were mostly higher at CK-1-3, indicating that they functioned downstream of sucrose catabolism and promoted the accumulation of RFOs. However, as the stress deepened (temperature decreased or time extended), the genes encoding functional enzymes such as PME, PG, BG, EG, and SUS were down-regulated, resulting in a large reduction of hexoses such as glucose, and the lower glucose content may lead to an increase in osmotic potential.

The products of sucrose metabolism generated fructose-6-phosphate (F6P) by the action of hexokinase, which was catalyzed by fructose 6-phosphate kinase (PFK) to generate fructose-1,6-bisphosphate (F-1,6-BP), which then entered the glycolytic process. During glycolysis, the expression of genes encoding glyceraldehyde-3-phosphate dehydrogenase (GAPDH) and ethanol dehydrogenase (ADH) differed among treatments. The expressions of the GAPDH (*VIT_01s0010g02460*) and ADH (*VIT_04s0044g01120, VIT_04s0044g01130*, *VIT_04s0044g01110*) genes were suppressed with increasing time (B vs. A, C vs. A), indicating that the glycolytic process was inhibited in cells under freezing stress, reducing energy consumption and possibly increasing the accumulation of reducing sugars to play an osmoregulatory role.

#### 2.4.3. Effect of Freezing Stress on Lipid Metabolism

**Figure 7:**
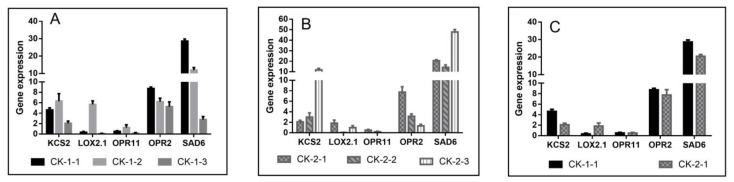
Expression of genes related to lipid metabolism. (**A**): Expression of common DEGs related to lipid metabolism at three time points under −10 °C treatment (CK-1-1 vs. CK-1-2 vs. CK-1-3); (**B**): Expression of common DEGs related to lipid metabolism at three time points under −16 °C treatment (CK-2-1 vs. CK-2-2 vs. CK-2-3); (**C**): Expression of common DEGs related to lipid metabolism at different temperatures for 3h (CK-1-1 vs. CK-2-1).

Low temperature also caused changes in lipids, and five common DEGs were detected between groups. 3-ketoacyl-CoA synthase (KCS) is a key enzyme in the ultra-long-chain monounsaturated fatty acid biosynthetic pathway, catalyzing the condensation of malonyl-CoA with lipoyl-CoA in the endoplasmic reticulum pathway to extend the fatty acid carbon chain. Freezing stress activated the expression of *KCS2*, which was up-regulated with increasing stress time, with the peak of *KCS2* expression occurring at 6 h under −10 °C stress (Figure 7A) and at 12 h under −16 °C stress (Figure 7B). 

The desaturation of long-chain fatty acids is accomplished in successive steps: Stearoyl-CoA Desaturase (SAD) adds the first double bond to saturated fatty acids. In this study, the expression of *SAD6* was consistently higher under low-temperature stress and gradually decreased with increasing stress duration at −10 °C stress (Figure 7A), while −16 °C showed an increase at 12 h (Figure 7B). A marked increase in the expression of *KSC2* and *SAD6* occurred at the later stage of −16 °C stress, probably due to the serious damage to the cell membrane of the plant at this time and the disturbance of lipid metabolism, so only the expression of each gene between the two temperatures at 3 h was compared (Figure 7C). The expression of both *KSC2* and *SAD6* under 16 °C stress was lower than that at −10 °C, indicating that the ability of plants to synthesize long-chain unsaturated fatty acids was reduced under extreme temperature, which may have led to a decrease in membrane fluidity and stability and the leaching of intracellular compounds.

Fourteen genes encoding lipoxygenase (LOX) were identified and observed, among which *LOX2.1* showed a difference at different times and different stress temperatures. LOX2 activity is repressed in intact cells but rapidly activated after tissue injury, which may account for the substantial increase in *LOX2.1* gene expression in CK-1-2 compared with CK-1-1 (Figure 7A) and the higher expression in CK-2-1 compared with CK-1-1 (Figure 7C). Up-regulation of *LOX2.1* may catalyze the conversion of α-linolenic acid to linolenic acid-9-hydroperoxide (9-HPOT) and linolenic acid-13-hydroperoxide (13-HPOT). In addition, 13-HPOT can be further metabolized by 12-oxygen phytodienoic acid reductase (OPR) to produce oxygenated lipids, such as jasmonic acid and its derivatives (JAs); 12-oxygen phytodienoic acid reductase-related genes (*OPR2, OPR11*) were similarly differentially expressed (Figure 7).

#### 2.4.4. Effect of Freezing Stress on Secondary Metabolite Synthesis

**Figure 8:**
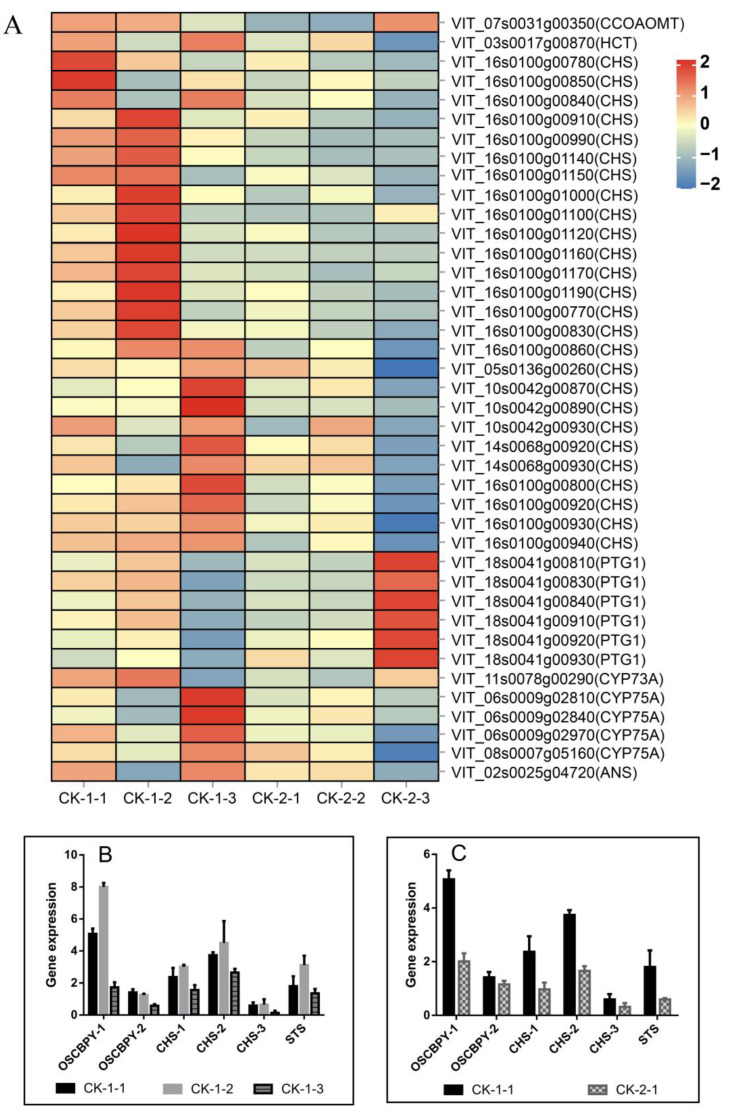
Expression of DEGs related to secondary metabolite synthesis. (**A**): Heatmap of DEGs expression in the flavonoid synthesis pathway under different treatments; (**B**): Expression of common DEGs related to secondary metabolites under different stress times (CK-1-1 vs. CK-1-2 vs. CK-1-3); (**C**): Expression of DEGs related to secondary metabolites under different stress temperatures (CK-1-1 vs. CK-2-1).

The flavonoid synthesis pathway was one of the metabolic pathways that differed most significantly between treatments (Table S1). A total of 22 differential genes were enriched in the flavonoid synthesis pathway between CK-1-1 vs. CK-1-2 vs. CK-1-3, affecting the activities of chalcone synthase (CHS), trans-cinnamic acid 4-monooxygenase (CYP73A), flavonoid 3’,5’-hydroxylase (CYP75A), anthocyanin synthase (ANS), and root bark glycoside synthase (PGT1). The genes encoding PGT1 and CYP73A and most of the genes encoding CHS had the highest expression at 6 h, promoting the synthesis of intermediates such as coumaroyl coenzyme A, chalcone, and rhizidine, while the genes encoding ANS and CYP75A showed the highest expression at 12 h, possibly catalyzing flavonoid synthesis downstream (Figure 8). Among them, the genes encoding CHS, *CHS-1* (*VIT_16s0100g01140*), *CHS-2* (*VIT_16s0100g00990*), and *CHS-3* (*VIT_16s0100g01150*), were the common DEGs under the three stress times (Table 1). The expression of *CHS-1*, *CHS-2*, and *CHS-3* increased under cold stress, but their expression was inhibited with increasing stress time (CK-1-2 vs. CK-1-3) and decreasing temperature (CK-1-1 vs. CK-2-1) (Figure 8). In addition, with the extension of the stress time to 12 h, CK-2-3 significantly decreased the expression of genes encoding mangiferyl-O-hydroxycinnamoyltransferase (HCT), CHS, and CYP75A compared with CK-1-3, thereby inhibiting the synthesis of various flavonoids such as downstream naringenin, quercetin, and popcornin, and the flavonoid pathway may be shifted more in the direction of rhizobioside synthesis. In addition to CHS, genes encoding stilbene synthase (STS) and β-amyloid synthase (OSCBPY) were also identified as common DEGs across comparison groups (Table 1). *STS* (*VIT_16s0100g00900*) and *OSCBPY-1* (*VIT_09s0054g01220*) were significantly up-regulated under freezing stress (CK-1-1 vs. CK-1-2), suggesting that that stilbenoids and triterpenoids may play a role in the low-temperature defense response of Cabernet Sauvignon (Figure 8).

## 3. Discussion

Many studies have revealed the transcription of grapes under low temperature, mainly focusing on the comparison between *V. vinifera* L. and *Vitis amurensis*, to assess the transcriptome differences between species with different cold-resistance phenotypes [36,37]. There are also some studies on bud [38], leaf [39], and flower [40] tissues that reveal the response of grape to low temperatures in the growing season. We focus on the response of dormant branch tissue to low temperature below the freezing point, aiming to provide a reference for the research of Eurasian grape under freezing stress.

### 3.1. Low-Temperature Signal Transduction

The response of plants to cold stress can be divided into several steps: cold signal perception and reception, signal transduction, response of upstream and downstream gene expression networks, and ultimately physiological and biochemical changes [41]. At present, it is not clear how plants sense low-temperature signals [42], but it is widely recognized that the level of cytoplasmic Ca^2+^ acts as the second messenger to transmit low-temperature signals. Five calcium receptor-related genes showed high expression at the early stage of freezing stress (Figure 5), indicating the important role of calcium/calmodulin-mediated signal transduction in plants’ response to cold stress. 

*CML8*, a common DEG, affects four pathways: plant–pathogen interaction, plant hormone signal transduction, the MAPK signal pathway—plant, and the phosphatidylinositol signal system. *CML8* is an important Ca^2+^ sensor, which has been reported to be involved in the plant defense response against *Pseudomonas syringae*, affecting the accumulation of PR1 and salicylic acid (SA) [43]. PR1 is a class of pathogen-related proteins (PRs), which are induced by pathogen infection, environmental stress, compounds, and trauma [44]. Goyal et al. [45] reported that low temperature could induce the accumulation of the *PR1b1* transcript and its protein in tomato fruit, and the accumulation of the PR1b1 protein continued to increase during the rewarming process after low-temperature treatment, which was also affected by the SA signal. The function of the PR1B1 protein is not clear—it may play a role as an antifreeze protein [46] or it may be a plant defense mechanism [45], but the plant–pathogen interaction pathway it affects has been reported to be related to the cold acclimation of grapes [47]. This study showed the upregulation of the *PR1B1* gene (Figure 5), which occurred later than *CML8* gene expression, indicating that it may be induced by *CML8*. Our study also identified seven MPK-related genes, but only *MPK9* showed differences between treatments, which may be related to cold signaling. *MPK9* can function downstream of ROS signaling to positively regulate Abscisic acid (ABA) signaling and regulate stomatal closure [48], and its high expression in CK-2-3 may be associated with elevated ROS concentrations (Figure 5).

### 3.2. Carbohydrate Metabolism

Carbohydrate metabolism is the center of all biological life activities and connects protein, lipid, nucleic acid, and secondary substance metabolism [49]. The regulation of gene expression related to carbohydrate metabolism enzymes may play a key role in the corresponding low-temperature stress of plants [50]. Low-temperature stress leads to the disorder of plant physiological metabolism, and the most serious is the ice damage of tissues. In this study, vines enhanced the rigidity of the cell wall through the hydrolysis of pectin to alleviate damage caused by ice. Bilska Kos et al. [51] reported that the activity of PME was first observed to increase under cold treatment and to then decrease with the increase in treatment time, and the PME activity decreased faster in cold-sensitive plants. Cold stress also greatly affected the expression of PG-related genes [40], and the difference before and after cold treatment in *Vitis amurensis* was more than 20 times [36]. On the one hand, the hydrolysates of pectin OGs provide carbon sources for intracellular carbohydrate metabolism; on the other hand, they can also act as signaling molecules to bind with cell wall-associated kinases (WAKs) to activate cell defense responses [52,53].

The degradation of cellulose also provides soluble sugar for cells, which can reduce the osmotic potential and freezing point of cytoplasm, inhibit the formation of ice nuclei, and maintain the stability of the cell structure. In the process of low-temperature-induced dehydration, some functions of water may be replaced by multiple hydroxyl groups of sugars in cells, thus protecting protein activity [49]. Low temperature will also lead to the reduction of the sucrose content, which is shown by stimulating the degradation of sucrose, increasing hexose phosphate, and limiting the synthesis of sucrose, which will help to improve cold resistance [54]. The metabolic products of sucrose, RFOs, accumulate under low temperature. RFOs can increase the water-holding capacity of cells as osmoregulation substances, stabilize the structure of cell membranes as cryoprotectants, assist in the removal of free radicals, and alleviate oxidative stress [55,56].

In addition to sugar conversion, the process of glycolysis also changed greatly at low temperatures. The expression of *GAPC2*, the gene encoding glyceraldehyde-3-phosphate dehydrogenase (GAPDH), was inhibited, which may lead to a large reduction of 3-PGA and a significant increase in DHA, F-1,6-BP, F6P, F6P, and other transformation metabolites, thus accumulating a large amount of reducing sugar. The change of these metabolite banks may be due to slowing of the turnover of glycolysis, so that sugar metabolism develops toward the direction of reducing sugar accumulation [57]. However, the reduction of ATP consumption may lead to excessive ROS, eventually leading to oxidative damage and even programmed cell death [58], which may be the cause of the mutation of some functional enzyme genes at CK-2-3. Programmed cell death leads to high degradation of cell wall components, and a large amount of soluble sugar is decomposed into ethanol. Glycolysis products such as GAP and PEP also provide precursors for the synthesis of lipids and secondary metabolites.

### 3.3. Membrane Damage and the Metabolism of Unsaturated Fatty Acids

Plants usually respond to low-temperature stress by increasing membrane lipid unsaturation, altering lipid composition, or changing lipid/protein ratios [59]. 3-ketoacyl-CoA synthase (KCS) is the key enzyme in the fatty acid biosynthetic pathway, catalyzing the extension of fatty acid carbon chains. The transcript level of *KSC* is influenced by environmental conditions and plays a role in osmotic stress, light stress, salt stress, and low-temperature stress [60,61]. In addition, low temperature induces an increase in fatty acid desaturase activity, resulting in a higher unsaturated fatty acid content, lower phase transition temperature of membrane lipids, better membrane fluidity and stability, and increased cold tolerance of plants [62]. Stearoyl ACP desaturase (SAD) can regulate the amount of unsaturated fatty acids and the ratio of saturated to unsaturated fatty acids in higher plants [63]. Previous studies have shown that overexpression of *ZmSAD1* decreases the content of stearic acid and the ratio of saturated to unsaturated fatty acids in maize [64].

In addition to the sclerosis of cell membranes, another aspect of low-temperature-mediated membrane damage involves oxidative damage by hydrogen peroxide [65]. Lipoxygenase (LOX) is widely present in higher plants and is associated with plant growth and development, senescence and fruit ripening, disease resistance, trauma response, and other stress responses [66]. It catalyzes the oxidation of unsaturated fatty acids, causes lipid peroxidation in plant cell membranes, forms volatile substances such as hydrogen peroxide derivatives, and catalyzes the production of malondialdehyde. The increase in relative conductivity and the rise in the MDA content at low temperature indicate membrane damage and may be related to the upregulation of *LOX2.1* (Figure 1).

The release of polyunsaturated fatty acids such as linolenic acid from plant membrane lipid degradation under the action of adversity factors is catalyzed by lipoxygenase (LOX) causing two different pathways, resulting in a series of bioactive compounds called oxylipins, including jasmonic acid and its derivatives, six-carbon volatiles, and 9-HPOT derivatives, all known to be important signaling molecules during plant stress responses [67,68,69,70,71]. 

### 3.4. Flavonoids and Stilbenes

The synthesis of flavonoids is influenced by environmental factors such as UV light, salt, high and low temperatures, and drought stress and is considered to be an important component of plant resistance to abiotic stresses [72,73,74,75,76]. In addition to scavenging ROS, flavonoids may also have a direct effect on the stability of cell membranes. Under freezing conditions, amphiphiles such as flavonoids may enter the hydrophobic phase of the membrane more strongly when most of the water in the cell becomes intercellular ice crystals [77,78]. It has been shown that the flavonoid biosynthetic pathway in Arabidopsis is strongly cold induced [79,80]. There is a significant correlation between the flavonoid content and freezing resistance [81]. Up-regulation of genes associated with dihydroflavonol and isoflavone synthesis was also observed in the cold acclimation of grapes [47]. Chalcones are common synthetic precursors of numerous flavones, flavanones, flavonols, flavanols, isoflavones, and anthocyanins [82]. In our study, up-regulation of most of the CHS genes occurred with increasing stress time at −10 °C, but an overall decrease in CHS gene expression was observed after the temperature dropped to −16 °C, suggesting that under extreme temperatures, the ability of vines to synthesize flavonoids and scavenge excess free radicals is inhibited and the cells may suffer severe oxidative damage. Stilbene is usually considered an effective plant antitoxin, which has bactericidal, anti-inflammatory, and other effects and participates in plant defense against pathogens [83,84,85]. The analysis of the *VpSTS* sequence showed that there was a low-temperature response element (LTR) in its promoter region, indicating that *STS* would be expressed under cold induction [85].

### 3.5. Hypothetical Model of Grapevine Responding to Low-Temperature Stress

**Figure 9:**
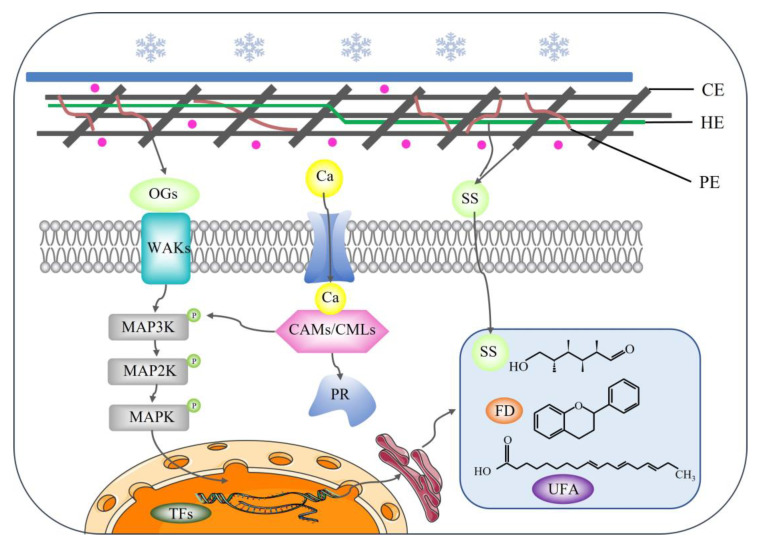
Hypothetical model of DEG expression events in grapevine responding to freezing stress. CE: cellulose; HE: hemicellulose; PE: pectin; OGs: oligogalacturonic acid; WAKs: cell wall-associated kinases; PR: pathogenesis-related protein; TFs: transcription factors; SS: soluble sugar; FD: flavonoids; UFA: unsaturated fatty acid.

Based on the comparison of the results of transcriptomic analysis under different stress conditions in Cabernet Sauvignon, a hypothetical model of the low-temperature response was developed (Figure 9). Under freezing stress, the increase in Ca^2+^ was first recognized by the Ca^2+^ receptor (CAM/CML), which activated the MAPK cascade reaction and caused changes in phosphorylation modifications, which were then regulated by each transcription factor and affected the synthesis of downstream metabolites. Plant pathogenesis-related protein 1 (PR1) may also play a role in downstream calcium signaling. Freezing stress also activated the expression of various cell wall enzyme-related genes, including pectinases (PME, PG, PL) and cellulases (BG, EG), promoting pectin and cellulose degradation, and the oligogalacturonides (OGs) produced by their degradation may act as signaling molecules bound to cell wall-associated kinases (WAKs), which also act on the MAPK cascade reaction. Hydrolysis of the cell wall also provides more soluble sugars in the cell, and downstream genes in sugar metabolism related to starch and sucrose catabolism (SUS, INV) and the synthesis of oligosaccharides of the cottonseed sugar series (GOLS, RFS) are upregulated, and some genes involved in the glycolytic process (GAPDH, ADH) are repressed, all favoring the accumulation of more reducing sugars for osmotic protection against cell dehydration and cell structure breakdown caused by low temperature. The specific effects on lipid metabolism were manifested by the activation of lipoxygenase gene (*LOX2.1*) expression to promote the metabolism of linolenic acid to downstream oxygenated lipids, as well as the activation of 3-ketoesteroyl-CoA synthase gene (*KCS2*) and stearoyl-ACP desaturase gene (*SAD6*) expression to increase the synthesis of long-chain unsaturated fatty acids in response to low-temperature stress. In addition, genes encoding chalcone synthase (CHS), stilbene synthase (STS), and β-amyloid synthase (OSCBPY) were upregulated to promote the accumulation of secondary metabolites, which may function as reactive oxygen scavengers or cell membrane protectors.

## 4. Material and Methods

### 4.1. Plant Materials and Freezing Treatment

This experiment was conducted at the Shengtang Winery, Yangling, China. Two-year-old cuttings of cv. Cabernet Sauvignon were used for the experiment. Grape seedlings were cultivated in 40 × 40 × 30 cm pots with a substrates composed of garden soil, perlite, and humus (2:1:1, volume ratio) and grown naturally in the field. The conventional cultivation management in the growing season is to keep 1–2 annual branches per vine according to the growth. After the branches grew to the height of 10 buds, the cores were picked and the secondary branches were removed. Unless there was rainfall, irrigation was conducted every 15 days without fertilization. Each treatment was set with 3 biological replicates and 3 vines per replicate. The 2nd and 3rd buds and internodes from the base of the branch of each vine were collected, and 0.5 g of sample was ground for RNA extraction.

After natural dormancy in December, the vines in the pots were transferred to the high- and low-temperature alternating test chamber (YSGJS-408, Shanghai Lanhao Instrument & Equipment Co., Ltd., Shanghai, China) for freezing stress treatment. The low-temperature treatment referred to the method of Han et al. [35], and the rate of cooling and rewarming was 4 °C/h. The branch samples for electrolyte leakage measured were treated at −6 °C, −8 °C, −10 °C, −12 °C, −14 °C, −16 °C, −18 °C, and −24 °C for 12 h and recovered at 4 °C for 12 h. The samples for transcriptomic analysis were treated at −10 °C and −16 °C for 3 h, 6 h, and 12 h, frozen in liquid nitrogen immediately and stored at −80 °C. The numbers of each treatment group are shown in Table 2.

### 4.2. Determination of Electrolyte Leakage

The relative electrical conductivity was determined according to the method of [35]. Samples (weighing 2.0 g) of branches were cut into 0.3–0.5-cm-thick slices and placed into a 25 mL test tube with a stopper. Next, 20 mL of distilled water was added, the tube was stoppered and then shaken in a shaker for 12 h before the conductivity (mS•cm^−1^) was measured (EL1) using a conductivity meter (DDS-307A, Shanghai optical instrument factory, Shanghai, China). The test tubes were then boiled for 40 min, allowed to stand for 2 h, and then the conductivity value was remeasured (EL2). The relative conductivity (RC) was calculated according to Equation (1):

(1) $$\mathrm{Relative}\;\mathrm{conductivity}\;\left(\mathrm{RC}\right)=\frac{\mathrm{EL}1}{\mathrm{EL}2}\times 100\%$$

### 4.3. Determination of the Malondialdehyde Content

The malondialdehyde (MDA) content was determined by thiobarbituric acid-reactive substances method [86]. Branch samples (0.5 g) were homogenized in 5 mL 0.1%TCA and then centrifuged at 5000 rpm for 10 min. Next, 5 mL of 5% TCA containing 0.5% TBA was added to 1 mL of the supernatant, followed by incubation in boiling water for 10 min and then transferred to ice water to stop the reaction. MDA absorption was measured spectrophotometrically at 450, 532, and 600 nm.

### 4.4. RNA Extraction, Library Construction, and Sequencing 

Samples were ground in liquid nitrogen, and 100 mg of powder was used for the total RNA extraction. Total RNA was extracted using a Trizol reagent kit (Invitrogen, Carlsbad, CA, USA) according to the manufacturer’s protocol. RNA quality was assessed on an Agilent 2100 Bioanalyzer (Agilent Technologies, Palo Alto, CA, USA) and checked using RNase-free agarose gel electrophoresis. After total RNA was extracted, mRNA was enriched by Oligo(dT) beads. Then, the enriched mRNA was fragmented into short fragments using fragmentation buffer and reverse transcribed into cDNA by using the NEBNext Ultra RNA Library Prep Kit for Illumina (NEB #7530, New England Biolabs, Ipswich, MA, USA). The purified double-stranded cDNA fragments were end repaired, A base added, and ligated to Illumina sequencing adapters. The ligation reaction was purified with AMPure XP Beads (1.0X). Ligated fragments were subjected to size selection by agarose gel electrophoresis and polymerase chain reaction (PCR) amplification. The resulting cDNA library was sequenced using an Illumina Novaseq6000 by GeneDenovo Biotechnology Co. (Guangzhou, China).

### 4.5. RNA-Seq Data Analysis

Reads obtained from the sequencing machines were further filtered by fastp (version 0.18.0) [87] to remove reads with adapters, reads containing poly-N (N content > 10%), and low-quality reads (Q-value < 20). The short reads alignment tool Bowtie2 (version 2.2.8) [88] was used for mapping reads to the ribosome RNA (rRNA) database. The rRNA mapped reads were removed. The remaining clean reads were further used in assembly and gene abundance calculation. Paired-end clean reads were mapped to the reference genome of ‘Pinot Noir’ using HISAT2. 2.4 [89] with “-rna-strandness RF” and other parameters set as default. The mapped reads of each sample were assembled by using StringTie v1.3.1 [90,91] in a reference-based approach. For each transcription region, an FPKM (fragment per kilobase of transcript per million mapped reads) value was calculated to quantify its expression abundance and variations using RSEM software [92].

RNA differential expression analysis was performed using DESeq2 software [93] between two different groups. The genes with a false discovery rate (FDR) below 0.05 and absolute fold change ≥2 were considered differentially expressed genes (DEGs). The GSEA and MSigDB software packages were used for enrichment analysis to identify whether a set of genes in specific GO terms/KEGG pathways differed significantly between two groups [94]. Trend analysis was performed by ShortTime-series Expression Miner software [95] with screening criteria of -pro 20 and -ratio 1.0000.

## 5. Conclusions

Under low-temperature stress, plants initiate a series of defense responses, a process that involves multiple cellular functions and metabolic pathways. In this study, we sequenced the transcriptome of cv. Cabernet Sauvignon under different stress temperatures and times and combined GO and KEGG enrichment analyses to explore the expression patterns of key genes at low temperatures. The results indicated that

(1) More genes were differentially expressed with increasing stress time, but the most common DEGs had peak expression at 6 h. The differential genes under different freezing treatments were mainly enriched in the following GO terms: metabolic processes, cellular processes, binding, catalytic activity, cells, cell parts, and membranes.
(2) Calcium/calmodulin-mediated signaling played a key role in the low-temperature response, most calcium receptors were rapidly activated in the early stages of freezing stress, and genes encoding PR1B1 proteins were up-regulated at low temperatures.
(3) Carbohydrate metabolism, lipid metabolism, and the synthesis of secondary metabolites were the main differential pathways for KEGG enrichment. The hydrolysis of pectin and cellulose in the cell wall may be an important strategy for grapevines to cope with freezing injury.

This study provides new insights into the molecular mechanisms of cold resistance in *V. vinifera* L., suggesting a hypothetical model for low-temperature response. Common differentially expressed genes may serve as candidate genes for germplasm improvement and evaluation.

## Figures and Tables

**Table 1:** (**A**) Kyoto Encyclopedia of Genes and Genomes (KEGG) enrichment analysis of common DEGs under different temperatures. (**B**) KEGG enrichment analysis of common DEGs at different times.

(**A**)				
Classification	Pathway ID	Pathway	Genes	Description
Signal Transduction	ko04626	Plant–pathogen interaction	VIT_07s0141g00060 (*KCS2*)	KCS; 3-ketoacyl-CoA synthase [EC:2.3.1.199]
	ko04712	Circadian rhythm—plant	VIT_16s0100g01140 (*CHS-1*)	CHS; chalcone synthase [EC:2.3.1.74]
			VIT_16s0100g00990 (*CHS-2*)	CHS; chalcone synthase [EC:2.3.1.74]
Lipid metabolism	ko00591	Linoleic acid metabolism	VIT_06s0004g01480 (*LOX2.1*)	LOX2S; lipoxygenase [EC:1.13.11.12]
	ko00592	Alpha-linolenic acid metabolism	VIT_06s0004g01480 (*LOX2.1*)	LOX2S; lipoxygenase [EC:1.13.11.12]
	ko00062	Fatty acid elongation	VIT_07s0141g00060 (*KCS2*)	KCS; 3-ketoacyl-CoA synthase [EC:2.3.1.199]
Carbohydrate metabolism	ko00040	Pentose and glucuronate interconversions	VIT_08s0007g07760 (*PG2*)	PG; polygalacturonase [EC:3.2.1.15]
			VIT_08s0007g07750 (*PG2*)	PG; polygalacturonase [EC:3.2.1.15]
	ko00520	Amino sugar and nucleotide sugar metabolism	VIT_05s0094g00360 (*EP3*)	chitinase [EC:3.2.1.14]
Amino acid metabolism	ko01230	Biosynthesis of amino acids	VIT_08s0007g05000 (*METK3*)	metK; S-adenosylmethionine synthetase [EC:2.5.1.6]
	ko00270	Cysteine and methionine metabolism	VIT_08s0007g05000 (*METK3*)	metK; S-adenosylmethionine synthetase [EC:2.5.1.6]
Biosynthesis of secondary metabolites	ko00941	Flavonoid biosynthesis	VIT_16s0100g01140 (*CHS-1*)	CHS; chalcone synthase [EC:2.3.1.74]
			VIT_16s0100g00990 (*CHS-2*)	CHS; chalcone synthase [EC:2.3.1.74]
	ko00945	Stilbenoid, diarylheptanoid and gingerol biosynthesis	VIT_16s0100g00900 (*STS*)	ST; stilbene synthase [EC:2.3.1.95]
	ko00909	Sesquiterpenoid and triterpenoid biosynthesis	VIT_09s0054g01220 (*OSCBPY-1*)	LUP4; beta-amyrin synthase [EC:5.4.99.39]
(**B**)				
**Classification**	**K_ID**	**Pathway**	**Gene**	**Description**
Signal Transduction	ko04626	Plant–pathogen interaction	VIT_03s0088g00690 (*PR1B1*)	PR1; pathogenesis-related protein 1
			VIT_00s0179g00280 (*CML8*)	CALM; calmodulin
	ko04075	Plant hormone signal transduction	VIT_03s0088g00690 (*PR1B1*)	PR1; pathogenesis-related protein 1
			VIT_19s0014g04690 (*GH3.6*)	GH3; auxin responsive GH3 gene family
	ko04016	MAPK signaling pathway-plant	VIT_03s0088g00690 (*PR1B1*)	PR1; pathogenesis-related protein 1
			VIT_00s0179g00280 (*CML8*)	CALM; calmodulin
	ko04070	Phosphatidylinos-itol signaling system	VIT_00s0179g00280 (*CML8*)	CALM; calmodulin
	ko04712	Circadian rhythm—plant	VIT_16s0100g01150 (*CHS-3*)	CHS; chalcone synthase [EC:2.3.1.74]
Lipid metabolism	ko01212	Fatty acid metabolism	VIT_18s0001g15460 (*SAD6*)	FAB2, SSI2, desA1; acyl-[acyl-carrier-protein] desaturase [EC:1.14.19.2 1.14.19.11 1.14.19.26]
	ko00061	Fatty acid biosynthesis	VIT_18s0001g15460 (*SAD6*)	FAB2, SSI2, desA1; acyl-[acyl-carrier-protein] desaturase [EC:1.14.19.2 1.14.19.11 1.14.19.26]
	ko01040	Biosynthesis of unsaturated fatty acids	VIT_18s0001g15460 (*SAD6*)	FAB2, SSI2, desA1; acyl-[acyl-carrier-protein] desaturase [EC:1.14.19.2 1.14.19.11 1.14.19.26]
	ko00592	alpha-Linolenic acid metabolism	VIT_18s0041g02060 (*OPR2*)	OPR; 12-oxophytodienoic acid reductase [EC:1.3.1.42]
			VIT_18s0041g02010 (*OPR11*)	OPR; 12-oxophytodienoic acid reductase [EC:1.3.1.42]
Carbohydrate metabolism	ko01200	Carbon metabolism	VIT_01s0010g02460 (*GAPC2*)	GAPDH, gapA; glyceraldehyde 3-phosphate dehydrogenase [EC:1.2.1.12]
			VIT_08s0058g01000 (*ASP1*)	GOT2; aspartate aminotransferase, mitochondrial [EC:2.6.1.1]
	ko00010	Glycolysis/Gluconeogenesis	VIT_01s0010g02460 (*GAPC2*)	GAPDH, gapA; glyceraldehyde 3-phosphate dehydrogenase [EC:1.2.1.12]
	ko00710	Carbon fixation in photosynthetic organisms	VIT_01s0010g02460 (*GAPC2*)	GAPDH, gapA; glyceraldehyde 3-phosphate dehydrogenase [EC:1.2.1.12]
	ko00500	Starch and sucrose metabolism	VIT_06s0004g01430 (*BGLU12*)	BG; beta-glucosidase [EC:3.2.1.21]
			VIT_16s0022g00670 (INV*DC4)	INV, sacA; beta-fructofuranosidase [EC:3.2.1.26]
	ko00040	Pentose and glucuronate interconversions	VIT_08s0007g07760 (*PG2*)	PG; polygalacturonase [EC:3.2.1.15]
			VIT_15s0048g00510 (*PECS-2.1*)	PE; pectinesterase [EC:3.1.1.11]
	ko00052	Galactose metabolism	VIT_16s0022g00670 (INV*DC4)	INV, sacA; beta-fructofuranosidase [EC:3.2.1.26]
Amino acid metabolism	ko01230	Biosynthesis of amino acids	VIT_01s0010g02460 (*GAPC2*)	GAPDH, gapA; glyceraldehyde 3-phosphate dehydrogenase [EC:1.2.1.12]
			VIT_06s0004g01430 (*BGLU12*)	BG; beta-glucosidase [EC:3.2.1.21]
			VIT_08s0058g01000 (*ASP1*)	GOT2; aspartate aminotransferase, mitochondrial [EC:2.6.1.1]
	ko00460	Cyanoamino acid metabolism	VIT_02s0033g00670 (*NIT4B*)	NIT4; beta-cyano-L-alanine hydratase/nitrilase [EC:3.5.5.4 4.2.1.65]
			VIT_02s0033g00770 (*NIT4B*)	NIT4; beta-cyano-L-alanine hydratase/nitrilase [EC:3.5.5.4 4.2.1.65]
	ko00270	Cysteine and methionine metabolism	VIT_08s0058g01000 (*ASP1*)	GOT2; aspartate aminotransferase, mitochondrial [EC:2.6.1.1]
Biosynthesis of secondary metabolites	ko00940	Phenylpropanoid biosynthesis	VIT_06s0004g01430 (*BGLU12*)	BG; beta-glucosidase [EC:3.2.1.21]
			VIT_18s0001g06850 (*PNC1*)	POD; peroxidase [EC:1.11.1.7]
			VIT_10s0003g05420 (*MEE23*)	K22395; cinnamyl-alcohol dehydrogenase [EC:1.1.1.195]
	ko01210	2-Oxocarboxylic acid metabolism	VIT_08s0058g01000 (*ASP1*)	GOT2; aspartate aminotransferase, mitochondrial [EC:2.6.1.1]
	ko00941	Flavonoid biosynthesis	VIT_16s0100g01150 (*CHS-3*)	CHS; chalcone synthase [EC:2.3.1.74]
	ko00909	Sesquiterpenoid and triterpenoid biosynthesis	VIT_10s0003g03650 (*OSCBPY-2*)	LUP4; beta-amyrin synthase [EC:5.4.99.39]

**Table 2:** The numbers of the transcriptome sequencing samples.

Stress Temperature/°C	Stress Time/h	Number
−10	3	CK-1-1
	6	CK-1-2
	12	CK-1-3
−16	3	CK-2-1
	6	CK-2-2
	12	CK-2-3

## Data Availability

Raw sequencing data of this article were deposited in the Sequence Read Archive database under accession number PRJNA924096 (https://www.ncbi.nlm.nih.gov/, accessed on 16 January 2023). Further inquiries can be directed to the corresponding author.

## Supplementary Materials

The following supporting information can be downloaded at https://www.mdpi.com/article/10.3390/ijms24043884/s1.
