# Supplementary material for: Transcriptomics Reveals the Effect of Short-Term Freezing on the Signal Transduction and Metabolism of Grapevine
Source: Int J Mol Sci. 2023 Feb 15;24(4):3884. doi: 10.3390/ijms24043884 (PMC9965549; doi:10.3390/ijms24043884)
Supplement: Supplementary file 1 [file ijms-24-03884-s001.zip › Supplementary Materials2.pdf]

Table S1 KEGG enrichment analysis of DEGs under different freezing treatments

Comparison groups	Pathway ID	Pathway	P-value
CK-1-1 vs CK-2-1	ko00040	Pentose and glucuronate interconversions	0.000
	ko00592	alpha-Linolenic acid metabolism	0.002
	ko04712	Circadian rhythm - plant	0.002
	ko01100	Metabolic pathways	0.004
	ko00941	Flavonoid biosynthesis	0.006
	ko01110	Biosynthesis of secondary metabolites	0.045
CK-1-2 vs CK-2-2	ko01100	Metabolic pathways	0.000
	ko04712	Circadian rhythm - plant	0.000
	ko00941	Flavonoid biosynthesis	0.000
	ko00040	Pentose and glucuronate interconversions	0.000
	ko01110	Biosynthesis of secondary metabolites	0.000
	ko00945	Stilbenoid, diarylheptanoid and gingerol biosynthesis	0.000
	ko00592	alpha-Linolenic acid metabolism	0.000
	ko00460	Cyanoamino acid metabolism	0.001
	ko00520	Amino sugar and nucleotide sugar metabolism	0.003
	ko00591	Linoleic acid metabolism	0.005
	ko00130	Ubiquinone and other terpenoid-quinone biosynthesis	0.022
	ko00073	Cutin, suberine and wax biosynthesis	0.026
CK-1-3 vs CK-2-3	ko00941	Flavonoid biosynthesis	0.000
	ko01110	Biosynthesis of secondary metabolites	0.000
	ko01100	Metabolic pathways	0.000
	ko04712	Circadian rhythm - plant	0.000
	ko00196	Photosynthesis - antenna proteins	0.000
	ko00010	Glycolysis / Gluconeogenesis	0.000
	ko00270	Cysteine and methionine metabolism	0.000
	ko01230	Biosynthesis of amino acids	0.001
	ko00360	Phenylalanine metabolism	0.001
	ko00350	Tyrosine metabolism	0.002
	ko00520	Amino sugar and nucleotide sugar metabolism	0.003
	ko00052	Galactose metabolism	0.004
	ko04075	Plant hormone signal transduction	0.008
	ko00920	Sulfur metabolism	0.013
	ko00053	Ascorbate and aldarate metabolism	0.014
	ko00950	Isoquinoline alkaloid biosynthesis	0.016
	ko00940	Phenylpropanoid biosynthesis	0.016
	ko04016	MAPK signaling pathway - plant	0.031
	ko00944	Flavone and flavonol biosynthesis	0.033
	ko00945	Stilbenoid, diarylheptanoid and gingerol	0.035

		biosynthesis	
	ko00480	Glutathione metabolism	0.036
	ko00592	alpha-Linolenic acid metabolism	0.037
	ko00960	Tropane, piperidine and pyridine alkaloid biosynthesis	0.038
	ko04141	Protein processing in endoplasmic reticulum	0.039
	ko00195	Photosynthesis	0.041
CK-1-1 vs CK-1-2	ko01100	Metabolic pathways	0.000
	ko00040	Pentose and glucuronate interconversions	0.000
	ko00592	alpha-Linolenic acid metabolism	0.001
	ko00591	Linoleic acid metabolism	0.001
	ko01110	Biosynthesis of secondary metabolites	0.001
	ko00460	Cyanoamino acid metabolism	0.001
	ko00350	Tyrosine metabolism	0.005
	ko00520	Amino sugar and nucleotide sugar metabolism	0.019
	ko00270	Cysteine and methionine metabolism	0.027
CK-1-1 vs CK-1-3	ko01110	Biosynthesis of secondary metabolites	0.000
	ko00196	Photosynthesis - antenna proteins	0.001
	ko00592	alpha-Linolenic acid metabolism	0.001
	ko00941	Flavonoid biosynthesis	0.002
	ko00460	Cyanoamino acid metabolism	0.003
	ko00071	Fatty acid degradation	0.004
	ko00350	Tyrosine metabolism	0.009
	ko04016	MAPK signaling pathway - plant	0.011
	ko01100	Metabolic pathways	0.022
	ko00010	Glycolysis / Gluconeogenesis	0.023
	ko00330	Arginine and proline metabolism	0.037
	ko00040	Pentose and glucuronate interconversions	0.038
	ko00270	Cysteine and methionine metabolism	0.046
	ko00920	Sulfur metabolism	0.049
CK-1-2 vs CK-1-3	ko01110	Biosynthesis of secondary metabolites	0.000
	ko00941	Flavonoid biosynthesis	0.000
	ko01100	Metabolic pathways	0.000
	ko04712	Circadian rhythm - plant	0.000
	ko00040	Pentose and glucuronate interconversions	0.000
	ko00052	Galactose metabolism	0.000
	ko00073	Cutin, suberine and wax biosynthesis	0.000
	ko00591	Linoleic acid metabolism	0.003
	ko00460	Cyanoamino acid metabolism	0.004
	ko00592	alpha-Linolenic acid metabolism	0.006
	ko00940	Phenylpropanoid biosynthesis	0.014
	ko00945	Stilbenoid, diarylheptanoid and gingerol biosynthesis	0.017
	ko00960	Tropane, piperidine and pyridine alkaloid	0.018

		biosynthesis	
CK-2-1 vs CK-2-2	ko00942	Anthocyanin biosynthesis	0.029
	ko00040	Pentose and glucuronate interconversions	0.000
	ko00591	Linoleic acid metabolism	0.001
	ko00592	alpha-Linolenic acid metabolism	0.015
	ko04075	Plant hormone signal transduction	0.029
	ko01100	Metabolic pathways	0.035
CK-2-1 vs CK-2-3	ko00040	Pentose and glucuronate interconversions	0.000
	ko00591	Linoleic acid metabolism	0.001
	ko00592	alpha-Linolenic acid metabolism	0.015
	ko04075	Plant hormone signal transduction	0.029
	ko01100	Metabolic pathways	0.035
CK-2-2 vs CK-2-3	ko01110	Biosynthesis of secondary metabolites	0.000
	ko00941	Flavonoid biosynthesis	0.000
	ko00940	Phenylpropanoid biosynthesis	0.000
	ko01100	Metabolic pathways	0.000
	ko00040	Pentose and glucuronate interconversions	0.001
	ko04712	Circadian rhythm - plant	0.001
	ko04016	MAPK signaling pathway - plant	0.002
	ko00360	Phenylalanine metabolism	0.002
	ko04075	Plant hormone signal transduction	0.005
	ko03030	DNA replication	0.007
	ko00920	Sulfur metabolism	0.010
	ko00945	Stilbenoid, diarylheptanoid and gingerol biosynthesis	0.011
	ko00520	Amino sugar and nucleotide sugar metabolism	0.013
	ko00350	Tyrosine metabolism	0.014
	ko00270	Cysteine and methionine metabolism	0.017
	ko00010	Glycolysis / Gluconeogenesis	0.017
	ko00071	Fatty acid degradation	0.023
	ko00910	Nitrogen metabolism	0.024
	ko00592	alpha-Linolenic acid metabolism	0.047
