# Supplementary figures and images for: Transcriptomics Reveals the Effect of Short-Term Freezing on the Signal Transduction and Metabolism of Grapevine
Source: Int J Mol Sci. 2023 Feb 15;24(4):3884. doi: 10.3390/ijms24043884 (PMC9965549; doi:10.3390/ijms24043884)

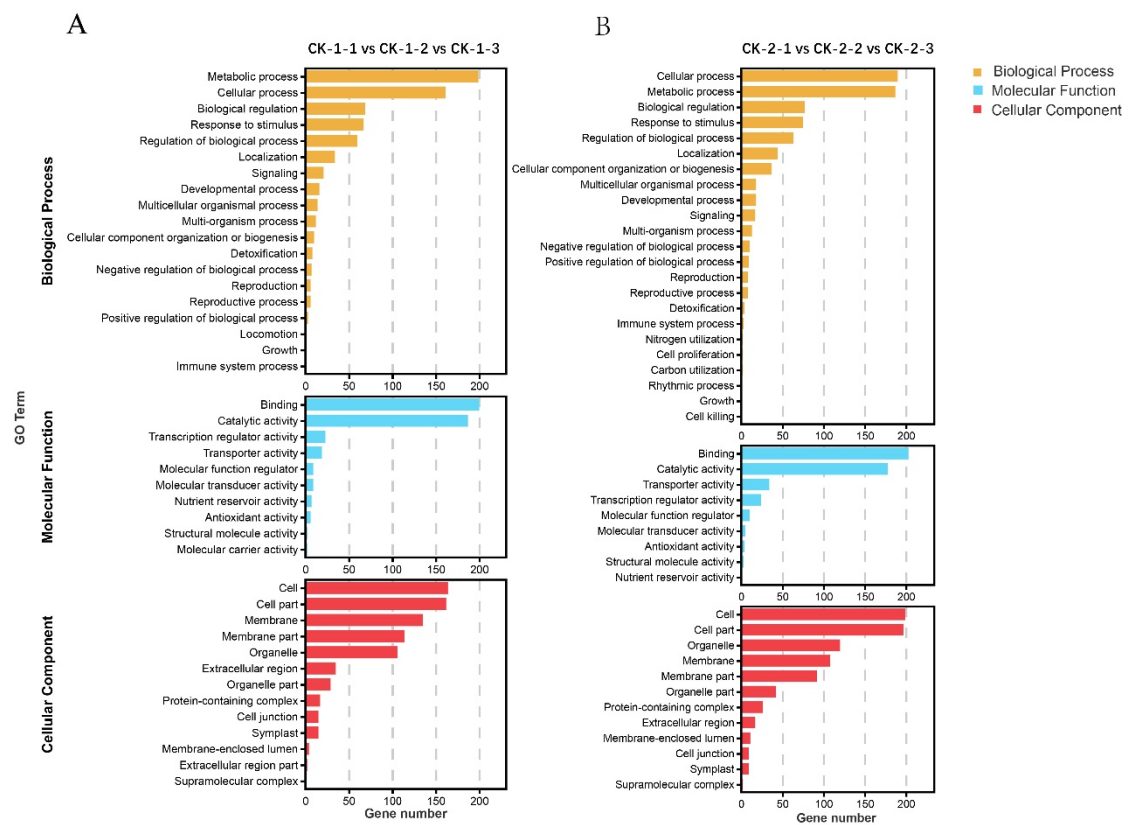

Figure S1 GO functional annotation analysis of DEGs in profile3 under different stress times

Supplement: Supplementary file 1 [file ijms-24-03884-s001.zip › Supplementary Materials1.pdf]
